# Acute Appendicitis Occurring in Dengue Fever: A Case Report and Review of the Literature

**DOI:** 10.1155/crdi/4654968

**Published:** 2025-07-21

**Authors:** Ayobamidele Rasaq Kolapo, Boluwatife Oluwafayoyimika Johnson

**Affiliations:** Department of Medicine, General Hospital Ikorodu, Ikorodu, Lagos, Nigeria

**Keywords:** acute abdomen, acute appendicitis, case report, dengue fever, dengue virus

## Abstract

Dengue fever is a disease caused by the dengue virus and it is present in many tropical countries, including Nigeria. Cases of acute abdomen have been reported in dengue fever and they include acute cholecystitis, acute pancreatitis, acute appendicitis, and nonspecific peritonitis. This case report illustrates a case of acute appendicitis in dengue fever and aims to describe the relationship between both conditions. We report a case of a 25-year-old lady who presented with symptoms of dengue fever and was confirmed to have the disease. She was on treatment for dengue but subsequently developed acute appendicitis. She later underwent appendectomy and the appendix was confirmed to be inflamed both intraoperatively and histologically. Dengue virus can cause periappendiceal inflammation (periappendiceal fluid collection and mesenteric lymphadenopathy/adenitis) or acute appendicitis (which can progress to appendiceal perforation or appendicular mass formation). Periappendiceal inflammation presents with clinical features mimicking acute appendicitis but without appendicitis on ultrasonography or laparoscopy. Periappendiceal inflammation can also progress to appendicitis. For patients with dengue fever, diagnosis of acute appendicitis should not be based on clinical findings alone. Serial ultrasonography to confirm appendicitis is recommended. Conservative treatment with antibiotics should be the first line for acute appendicitis in dengue fever or periappendiceal inflammation in dengue. Surgical treatment is required in cases of appendiceal perforation or failure of conservative management.

## 1. Introduction

Dengue fever is the most prevalent arboviral disease globally, and it is endemic in many tropical and subtropical regions of the world [1]. The disease is present in 128 countries with about 390 million cases annually [2]. Dengue fever is prevalent in many regions of Africa, with over 23 African countries having confirmed cases [2]. The disease was first reported in Nigeria in Ibadan in 1960, and there have been many reports of isolated dengue fever outbreaks in Nigeria following that [2, 3].

Dengue fever may present as asymptomatic disease, mild febrile illness, classic dengue fever, or severe dengue fever [4–6]. The severe disease could either be dengue hemorrhagic fever (DHF) or dengue shock syndrome (DSS) [6]. The mild and classic forms of the disease are usually self-limiting and resolve in less than 2 weeks. Patients with classic dengue present with high fever, headache, rash, arthralgia, myalgia, retro-orbital pain, nausea, and vomiting [5, 7].

Abdominal pain is one of the symptoms of classic dengue and has been described as one of the warning signs associated with increased risk of progression to severe disease [5]. Other abdominal features which are also associated with higher risk of severe dengue include vomiting, persistent vomiting, abdominal tenderness, ascites, gastrointestinal bleeding, and hepatomegaly. Acute abdomen, defined as sudden onset of progressive abdominal pain as the main feature of the patient's illness along with a peritoneal sign (tenderness, rebound tenderness, or guarding), has been reported to occur in dengue fever although in a much fewer proportion of patients than those with abdominal pain [1, 8]. The earliest reports of acute abdomen in patients who were later confirmed to have dengue fever were thought to be caused by an unusual presentation of dengue fever mimicking acute abdominal conditions [1, 4, 7, 9]. However, in recent years, there have been confirmed cases of acute abdominal conditions in dengue fever [6, 8, 10–15]. The commonest of these conditions in dengue infection are acute cholecystitis, acute pancreatitis, acute appendicitis, and nonspecific peritonitis [8, 10].

We report a case of a patient who developed acute appendicitis while on treatment for dengue fever.

## 2. Case Presentation and Management

A 25-year-old lady with no previous medical history presented to a secondary healthcare facility in Lagos, Nigeria, with fever, generalized body pains, and vomiting of 5 days duration. There was associated history of headache, generalized joint pains, muscle pains, body weakness, sore throat, chills, rigors, anorexia, painful swallowing, and passage of melena stool. There was no history of petechial rash, cough, or drenching night sweats.

Physical examination at presentation showed an acutely ill-looking lady with fever (T-38.3°C) and tenderness in the neck and upper limbs. There were no signs of meningeal irritation. Admitting blood pressure and pulse rate were 76/60 mmHg and 127 beats per minute, respectively. Abdominal examination revealed mild tenderness only in the epigastric region. There were no signs of abdominal organ enlargement, appendicitis, or peritonitis. Examinations of other systems were unremarkable.

Malaria parasite test (thick and thin film for microscopy) was negative. Full blood count done revealed hematocrit of 31%, leukocytopenia with white cell count of 3600/mm^3^, and thrombocytopenia with platelet count of 130,000/mm^3^. White cell differentials showed neutrophilia (N-79%), lymphocytopenia (L-8%), and monocytosis (M-13%). HIV screening, hepatitis B surface antigen, and hepatitis C virus were all negative. Serum electrolytes, urea, and creatinine showed hyponatremia (130.5 mmol/L) while other parameters were normal. Abdominopelvic ultrasound scan showed normal sonographic findings.

A presumptive diagnosis of acute febrile illness (KIV dengue fever) and acute gastritis was made. She was commenced on intravenous fluids, intravenous ceftriaxone 1 g 12 hourly, metronidazole 500 mg 8 hourly, paracetamol 1 g 8 hourly, omeprazole 20 mg 12 hourly, and amino acids–multivitamins infusion. Reverse transcriptase-polymerase chain reaction (RT-PCR) test for dengue virus was done and it came back positive, confirming the diagnosis.

Table 1 compares the clinical and laboratory features of this patient with those of traditional dengue.

Her clinical condition improved significantly on the above treatments. Body pains reduced and she began tolerating well orally. However, the fever persisted.

On the 8th day of admission, she began having severe pain in the right iliac fossa. Abdominal examination revealed certain findings, which have been outlined in Table 2.

An assessment of acute appendicitis was made. Since the diagnosis of acute appendicitis is clinical, she did not have a repeat ultrasonography or diagnostic laparoscopy done. She was then worked up for an emergency appendectomy. Repeat full blood count parameters are outlined in Table 3. Repeat serum electrolytes, urea, and creatinine were normal.

She subsequently had open appendectomy done under general anesthesia. Intraoperatively, the appendix was identified and seen to be inflamed. The appendix was 7 cm long and 1 cm in its widest diameter. It was then excised and sent for histology. She was transfused with 2 units of blood and 1 unit of 4% isoplasma (a synthetic colloid blood volume expander) intraoperatively.

Histopathology of the appendix showed transmural neutrophilic infiltration and edema, typical of acute appendicitis.

Postoperative period was uneventful. She was placed on intravenous fluids, antibiotics, and analgesics; and was commenced on oral intake after moving bowel. She was discharged home 6 days after the surgery and was followed up at the outpatient clinic. As of the time of writing this report, the patient is 6 months postsurgery. She is doing fine, has no symptoms, and has been discharged from the outpatient clinic.

Table 4 compares traditional dengue treatment with the treatment modalities used for this patient.

## 3. Discussion

Acute appendicitis occurring in dengue fever is rarer when compared with other acute abdominal conditions. Studies on acute abdomen in dengue have placed the incidence of acute appendicitis in dengue to be between 0.2% and 2% [6, 8].

The link between dengue fever and acute appendicitis has been a topic for debate. In one case series [4], a patient with confirmed dengue fever initially presented with symptoms and signs of acute appendicitis and had appendectomy done but there was no inflammation of the appendix seen at surgery, and only a small collection of serous fluid was seen around the right iliac fossa. Furthermore, in the same series, abdominal ultrasound scan done in seven patients with dengue fever who presented with clinical features of acute appendicitis revealed a collection of fluid around the appendix in five of these patients. In two other reports [7, 9] of patients who presented with clinical features of acute appendicitis but were later confirmed to have dengue fever, abdominal ultrasonography revealed fluid collection in the right iliac fossa but the appendix was not identified. Each patient later had appendectomy done and intraoperatively, a normal appendix was seen with multiple enlarged mesenteric lymph nodes.

The various reports of free fluid and mesenteric lymphadenopathy/adenitis around the appendix suggest that dengue virus causes periappendiceal inflammation [4]. The mechanism by which the virus does this is unclear; however, dengue virus is known to cause lymphoid hyperplasia/lymphadenopathy [4, 16], which explains the enlarged mesenteric lymph nodes. Furthermore, plasma leakage and serous effusion containing high protein content which is known to occur in DHF might play a role [1, 8]. The periappendiceal inflammation could explain the symptoms and signs of acute appendicitis in patients with dengue fever who were later confirmed not to have appendicitis and in whom mimicry was concluded [1, 4, 7, 9].

There are also multiple reports of confirmed acute appendicitis in dengue [6, 10]. In one report [11], a patient presented with clinical features of acute appendicitis and had appendectomy. Intraoperatively, the appendix was seen to be inflamed. Histology of the resected appendix showed transmural neutrophil infiltration, typical of acute appendicitis. Postoperatively, he developed thrombocytopenia and neutropenia, leading to the suspicion of DHF, which was then confirmed by testing. In a recent report by Thadchanamoorthy et al. [12], two patients developed clinical features of acute appendicitis during the recovery phase of DHF and appendicitis was confirmed intraoperatively and histologically. This was similar to what happened in our patient, who developed symptoms and signs of acute appendicitis while recovering from dengue fever.

It, therefore, appears that the effect of the dengue virus on the appendix could range from periappendiceal inflammation without appendicitis to acute appendicitis. Acute appendicitis, if not properly treated, could progress to appendiceal perforation or appendicular mass formation. There have been reports of appendiceal perforation in dengue fever [13] and appendicular mass formation in dengue fever [14]. One patient with dengue was intraoperatively discovered to have both appendiceal perforation and appendicular mass [15].

The pathophysiology of how periappendiceal inflammation (periappendiceal fluid collection and mesenteric lymphadenopathy/adenitis) progresses to acute bacterial appendicitis is not fully understood. It is believed to be due to viral invasion of the appendicular wall, which can cause edematous changes [8]. Plasma leakage and serous effusion into the appendicular wall caused by dengue virus can also contribute to the edematous changes [8]. Edematous changes can cause luminal obstruction, which can lead to secondary bacterial infection and appendicitis [10, 12]. The appendix contains abundant lymphoid tissue, thus lymphoid hyperplasia caused by the dengue virus can also play a role in luminal obstruction [12].

Concurrent bacteremia might be contributory as there could be hematogenous seeding to the already engorged appendix. Risk factors for concurrent bacteremia in dengue include older patient, prolonged fever (> 5 days), acute renal failure, gastrointestinal bleeding, altered consciousness, unusual dengue manifestation, and DSS [17]. Our patient in this report had prolonged fever and gastrointestinal bleeding. Bacterial infections that often coexist with dengue include *Escherichia coli*, *Staphylococcus aureus*, *Streptococcus species*, *Klebsiella pneumoniae*, *Pseudomonas aeruginosa*, *Acinetobacter baumannii*, and *Salmonella typhi* [18], and some of these organisms are known to be involved in acute appendicitis.

In one of the cases reported by Thadchanamoorthy et al. [12], three abdominal ultrasound scans done at different times revealed different findings. The first scan revealed only pericolic fluid collection, and the second scan done 5 days later revealed multiple enlarged mesenteric lymph nodes but the appendix was not visualized. The third scan then revealed acute appendicitis which had ruptured. This suggests that periappendiceal fluid collection occurs before mesenteric lymphadenopathy/adenitis, which occurs before appendicitis.

In some of the aforementioned reports, histology of the resected appendices in cases who underwent appendectomies revealed lymphocytic infiltrations or lymphoid follicular hyperplasia [1, 4, 8] while some had transmural neutrophil infiltration, which is typical of acute bacterial appendicitis [10–12, 15]. It is probable that in the former cases, the effects of the virus on the appendix were still at the point of viral-induced lymphoid hyperplasia/lymphocytic infiltrations and there was yet to be secondary bacterial infection. In our patient in this report, microscopy of the appendix showed transmural neutrophilic infiltration and edema. More studies to describe the pathogenesis of dengue virus in the appendix at the tissue level would be highly beneficial.

One population-based cohort study [5] done in Taiwan claimed that there is no increased risk of acute appendicitis among patients with dengue infection. However, this single study is not enough basis to dismiss the relationship between dengue fever and acute appendicitis, especially considering the multiple reports of acute appendicitis in dengue fever from different parts of the world. More studies are required to characterize the relationship between the two conditions.

In patients with confirmed dengue fever, diagnosis of acute appendicitis should not be based on clinical findings alone, as the patient might only be having periappendiceal inflammation. We recommend serial (daily) abdominal ultrasonography to confirm whether or not there is appendicitis and to monitor the disease progression. In confirmed cases of acute appendicitis in dengue without perforation, conservative treatment with intravenous antibiotics is recommended. Conservative treatment has been reported to be effective [6, 8, 10, 14]. Antibiotics treatment should also be done prophylactically for suspected periappendiceal inflammation as there is risk of progression to acute appendicitis. Following successful conservative treatment of appendicitis, the patient may be booked for interval appendectomy at a later time.

Failure of conservative treatment of appendicitis would require appendectomy. However, the point at which conservative treatment would be deemed to have failed is not clearly defined in literature. The clinician should combine the clinical course of the patient after antibiotics treatment for some days with the serial ultrasonography findings to determine the need for surgical intervention. For appendiceal perforation (detected on ultrasonography, plain x-ray, or computed tomography), surgical intervention should be done as the resulting peritonitis is a surgical emergency [13]. However, patients with dengue fever should be optimized before surgery as there is risk of excessive bleeding due to thrombocytopenia and coagulopathy that occurs in dengue [1, 8].

Asides the dengue virus, other viruses that have been implicated in acute appendicitis include measles virus, influenza virus, adenovirus, human immunodeficiency virus (HIV), human herpes virus, and rotavirus [19]. Therefore, a better understanding of the pathophysiology of dengue virus in acute appendicitis and its management could also have broader implications for managing these other viral infections.

## 4. Conclusion

It is important to recognize that acute appendicitis can occur as a complication of dengue fever as seen in our patient in this report. Diagnosis of acute appendicitis in dengue should not be based on clinical judgment alone but patients should have serial ultrasonography done. Acute appendicitis in dengue fever should be managed conservatively, but surgical treatment can be done in cases of perforation or failure of conservative management. Furthermore, patients in a dengue-endemic setting who present with clinical features of acute appendicitis and a full blood count showing thrombocytopenia/leukocytopenia should be evaluated for dengue fever as they might not necessarily require surgery.

## Figures and Tables

**Table 1:** Comparison of this patient's clinical and laboratory features with those of traditional dengue.

S/N	Feature	Traditional dengue	This patient
1	Fever	Present	Present
2	Vomiting	Present	Present
3	Headache	Present	Present
4	Arthralgia	Present	Present
5	Myalgia	Present	Present
6	Retro-orbital pain	Present	Absent
7	GI bleeding	Present	Present
8	Petechial rash	Present	Absent
9	Hepatomegaly	Present	Absent
10	Thrombocytopenia	Present	Present
11	Leukocytopenia	Present	Present
12	High hematocrit	Often present	Absent
13	Monocytosis	Often present	Present
14	Hyponatremia	Often present	Present

**Table 2:** Abdominal examination findings on Day 8 of admission.

S/N	Abdominal sign	Present/absent
1	Tenderness	Present (right hypochondriac, right inguinal, left inguinal regions)
2	Blumberg sign (rebound tenderness)	Present
3	Guarding	Absent
4	McBurney sign	Present
5	Rovsing sign	Present
6	Psoas sign	Present

**Table 3:** Repeat full blood count on Day 8 of admission.

S/N	Parameter	Value
1	Hemoglobin conc	8 g/dL
2	Hematocrit	24%
3	Platelet count	184,000/mm^3^
4	White cell count	7100/mm^3^
5	Neutrophils (percentage)	41%
6	Lymphocytes (percentage)	41%
7	Monocytes (percentage)	18%
8	Eosinophils (percentage)	0%
9	Basophils (percentage)	0%

**Table 4:** Comparison of treatment modalities used in traditional dengue with those used in this patient.

S/N	Treatment modality	Traditional dengue	This patient
1	Intravenous fluids	Used	Used
2	Antipyretics/analgesics	Used	Used
3	Surgical intervention (appendectomy)	Not used	Used
4	Blood transfusion	Sometimes used	Used
5	Synthetic colloid blood volume expander	Sometimes used	Used

## Data Availability

Data sharing is not applicable to this article as no new data were created or analyzed in this case report.
